# Consumer animal welfare and healthy perception of fresh sausages’ fiber fat replaced and elaborated with meat from non‐castrated male pigs

**DOI:** 10.1002/fsn3.4278

**Published:** 2024-07-04

**Authors:** Macarena Egea, M. Dolores Garrido, Maria Font‐i‐Furnols, Nuria Panella‐Riera, M. Belén Linares, Irene Peñaranda

**Affiliations:** ^1^ Department of Food Science and Technology, Veterinary Faculty University of Murcia Murcia Spain; ^2^ IRTA‐Food Quality and Technology Program, Finca Camps i Armet Monells Girona Spain

**Keywords:** castration, consumers, fiber, pork sausages

## Abstract

Although replacement of fat with fiber is a good strategy to avoid boar taint on high‐fat content products, as sausages, the final purchase intention is related to other external factors such as welfare and healthiness. So, the aim of this study was to evaluate the consumers' perception of sausages with fat replaced by fiber, elaborated with meat from non‐castrated male pigs, and to determine the influence of the consumers' habits and demographic parameters on their beliefs related to pig castration. An online survey was carried out. A total of 131 consumers answered a questionnaire about habits and beliefs related to meat and meat product issues. Subsequently, they evaluated four images of labeled products (castrated–not castrated/normal–reduced‐fat/traditional–high fiber content) with the Check‐all‐that‐apply (CATA) test. In all, 87.7% of meat consumers perceived meat products as positive, although 71% trimmed the fat previous consumption. In a 9‐point agreement scale, “Animal welfare worries me” obtained 7.5 and “the castration without anaesthesia should not be performed” 7.4. There was a higher grade of agreement with “castration of pigs justification to improve smell a flavour” in primary education level and in rural residence (*p* < .05). Sausages from castrated animals were perceived as fatty, juicy, appetizing, and animal cruelty. Reduced‐fat and rich/high fiber sausages were not associated with “healthy” but with “expensive” and “unpleasant.” Although reduced‐fat entire male pork sausages with vegetable fiber could be a better option for marketing uncastrated male pork, they will need more than fiber claims to be associated with healthy products and the consumer should be previously informed if he could appreciate the meaning of castration labeling.

## Graphical abstract

Entire male pork sausages reduced in fat and with vegetable fiber are positively perceived by consumers. Reduced‐fat entire male pork sausages with vegetable fiber can be an option for marketing this meat. Consumers consider castration to be justified if it improves the sensory perception of entire male pork.

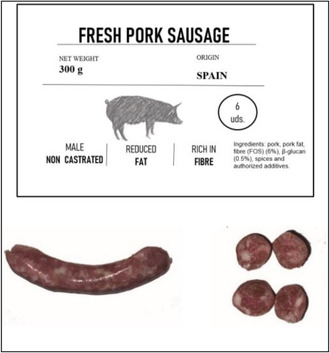

## 1 INTRODUCTION

The origin of food and respect for animals are some of the aspects that concern consumers. These issues have recently gained a lot of space in public discussion, not only for specific consumers, such as vegans and vegetarians, but also for consumers in general (Pugliese et al., 2023). In this sense, animal welfare is important when buying or consuming meat and sausages (De Araújo et al., 2022). Attitudes and expectations are mainly influenced by information about the characteristics of the process, beliefs and feelings, but the consumer's final decision also involves the taste of the meat, whether it is healthy or not, and the convenience of buying and preparing it. Consumers are supposed to consider a compromise between animal welfare, food quality, and food safety when purchasing pork (Aluwé et al., 2020).

European Union's (EU's) policy is clearly oriented toward improving animal welfare, and one of its main objectives is the welfare of pigs and the cessation of castration (Directive 2010/63/EU, 2010). As a consequence, non‐castrated pigs may accumulate boar taint. “Boar taint” is a sensory defect of meat related to puberty in animals, which develop an abnormal aroma and taste that some consumers may perceive when cooking or eating pork due to the accumulation of two substances (androstenone and skatole) in the fat of male pigs (Egea et al., 2020). Many consumers describe this odor as ammonia‐like, similar to that of urine or sweat, with a pungent and bitter taste (Iniesta et al., 2023). In order to reduce the perception of this odor and flavor, several strategies have been studied in fresh and transformed meat, such as smoking, curing, fermentation, or the addition of spices (Linares et al., 2022; Škrlep et al., 2020). However, these strategies could not be enough for meat products when animals have high levels of androstenone or skatole, since they tend to have a high‐fat content (20%–30%), and therefore, a high content of these compounds, due to their lipophilic nature (Peñaranda et al., 2023). In addition, cooked fresh products are usually consumed warm, which increases highly the probability of a negative sensory experience (Škrlep et al., 2020). An effective alternative for highly tainted entire male carcasses is blending. For meat patties (containing 30% fat tissue, spiced with pepper and added breadcrumbs), up to 40% of tainted meat (corresponding to 0.4 ppm (parts per million) androstenone in the product) was reported to be acceptable (Mörlein et al., 2019). Meat and meat products can suffer from negative consumer opinions associated with their unhealthy nutritional profile and high calorie content. Therefore, it is necessary to consider different strategies to offer healthier products and counteract these negative opinions, such as by reducing the fat content in meat or by increasing the amount of monounsaturated fatty acids (MUFAs) and polyunsaturated fatty acids (PUFAs) (Guedes‐Oliveira et al., 2021). However, fat is the main component of meat that contributes to its texture, flavor, and juiciness; thus, a reduction in fat content may only delay a decrement in characteristics, such as cooking performance, texture, and sensory perception (Bis‐Souza et al., 2020). Therefore, it may be important to replace fat with other ingredients that can fulfill a similar function and meet consumer demands (Peñaranda et al., 2023). One way to improve the nutritional profile of meat products is implemented by including probiotics, natural antioxidants, and vegetable fibers in the composition of the products (Guedes‐Oliveira et al., 2021). Therefore, the reformulation of meat must be considered. Vegetable fibers have been the main products chosen to replace fat in meat products (Egea et al., 2020). These vegetable fibers have been selected considering the technological properties and consumer health benefits they provide (Peñaranda et al., 2023). Previous studies have demonstrated that the reduction of fat in pork products elaborated with meat from non‐castrated male pigs is a good strategy to reduce boar taint perception (Egea et al., 2020; Peñaranda et al., 2023). A trained panel evaluated the reduction of fat in fresh Spanish and Frankfurt sausages elaborated with meat from non‐castrated male pigs (6.25 μg/g androstenone and 0.4451 μg/g skatole), in which fat was replaced with plant fibers (inulin, β‐glucan, and grape skin). The study concluded that plant fibers could offer a good strategy to mask boar taint (reduction of boar taint perception was up to 87.3%) and to provide a texture similar to that of commercial sausages (Egea et al., 2020). The replacement of pork fat with vegetable fibers, such as fructooligosaccharides (FOS) or β‐glucan, has been shown to have a positive effect on the texture of low‐fat meat products (Guedes‐Oliveira et al., 2021). In addition, they are considered prebiotic agents due to their functional effects on the gastrointestinal microbiota (Egea et al., 2020), so they could improve the nutritional profile of meat products.

Consumer perception of meat products is not only associated with physical and chemical composition, but nutritional quality, sensory properties, and social, ethical, or religious aspects could also influence purchase intention (Teixeira & Rodrigues, 2021). Any insight into consumers’ beliefs and attitudes is important, as they may contribute to behavioral intentions (Tomasevic et al., 2020). Studies on the affective and perceptual representations are interesting for the development and characterization of new products (Vidal et al., 2020). Therefore, the aim of this study was to evaluate the consumers' perception of sausages with fat replaced by fiber, elaborated with meat from non‐castrated male pigs, and to determine the influence of the consumers' habits and demographic parameters on their beliefs related to pig castration.

## 2 MATERIALS AND METHODS

### 2.1 Data collection

An online survey was designed in the Spanish language using the web‐based “Encuestas UMU” platform, a tool from the University of Murcia (UMU) questionnaire generation and management service. This survey was available from May 28 to June 13, 2021, and was disseminated through different social networks, such as WhatsApp, Facebook, and email, to consumers who lived in Spain, mainly in the Region of Murcia. Participants were not economically rewarded. Participation in the survey was voluntary and completely anonymous. Previous to the start, participants were informed that the survey was conducted by the University of Murcia for research purposes. The recruitment of participants was carried out trying to mimic the Spanish National population distribution by sex and age (INE, 2020). A total of 131 surveys were studied. The inclusion criterion of the consumers was that they should consume meat products (at least once a week).

### 2.2 Design and development of the questionnaire

The questionnaire consisted of four independent parts. Questions were selected from a previous bibliographical review of consumer studies on animal welfare and castration of pigs, some of them with some modifications. The questions and tests are presented in Table 1, together with the reference of the works from which they were obtained.

**TABLE 1:** Questionnaire design.

Section	Question	Answer	Reference
Demographic data	Gender	Female–male	Holman et al. (2017)
	Age (years)	18–30 31–45 46–60 More than 60	
	Area of residence	Rural Urban	Van Loo et al. (2014)
	Level of education	Primary Secondary Higher/university	
	Employment	Inactive Active Retired	
Consumption habits and experiences	Do you eat meat?		
	Have you had any unpleasant experiences consuming pork or meat products in the last six months?		Rimal (2005), Reinoso (2020)
	Do you usually read the information on the food labels?	Yes–no	
	Is your perception of pork products positive?		
	Do you trim fat from meat products or meat before eating?		
	Do you normally consume products rich in fibre?		
Consumer beliefs	Animal welfare worries me		Aluwé et al. (2020), Cabana (2010)
	I know that sometimes male pigs intended for consumption are castrated without anaesthesia at birth		
	I think castration of pigs without anaesthesia should not be done	Scale of 1–9 points	
	The castration of pigs is justified because it improves the smell and flavour of the meat		
	I believe that meat from castrated pigs should be labelled as such.		
	A high fat content in a meat product is negative for me		
CATA test	Check‐all‐that‐apply in the questionnaire	Dark, bright, animal cruelty, dry, fatty, light, matte, healthy, juiciness, appetizing, little fat, unhealthy, expensive, animal friendly, unpleasant, cheap	Aluwé et al. (2020), Egea et al. (2020)
Previous knowledge	Do you have knowledge about pigs castration?	Yes/no	

#### 2.2.1 Demographic data

The respondent had to answer basic demographic questions (gender, age, area of residence, level of education, and employment).

#### 2.2.2 Previous knowledge

An extra question regarding previous knowledge about castration was added at the end of the questionnaire to not influence the results: “Do you have knowledge about pigs castration?” with a yes–no answer.

#### 2.2.3 Consumption habits and experiences

This section consisted of six general questions about the habits and experiences of eating pork. Consumers were asked about their habits of meat consumption and the consumption of products rich in fiber, and also about their experience and perception when eating pork products. The answers were dichotomous, either “yes” or “no.”

#### 2.2.4 Consumer beliefs

A structured questionnaire was developed, which included five statements (beliefs) about castration and perception of the welfare of castrated pigs, and one more about fat content. The respondents had to indicate their degree of agreement with each statement according to a 9‐point Likert scale ranging from 1: “I totally disagree” to 9 “I totally agree.”

#### 2.2.5 CATA test (check‐all‐that‐apply)

**FIGURE 1:**
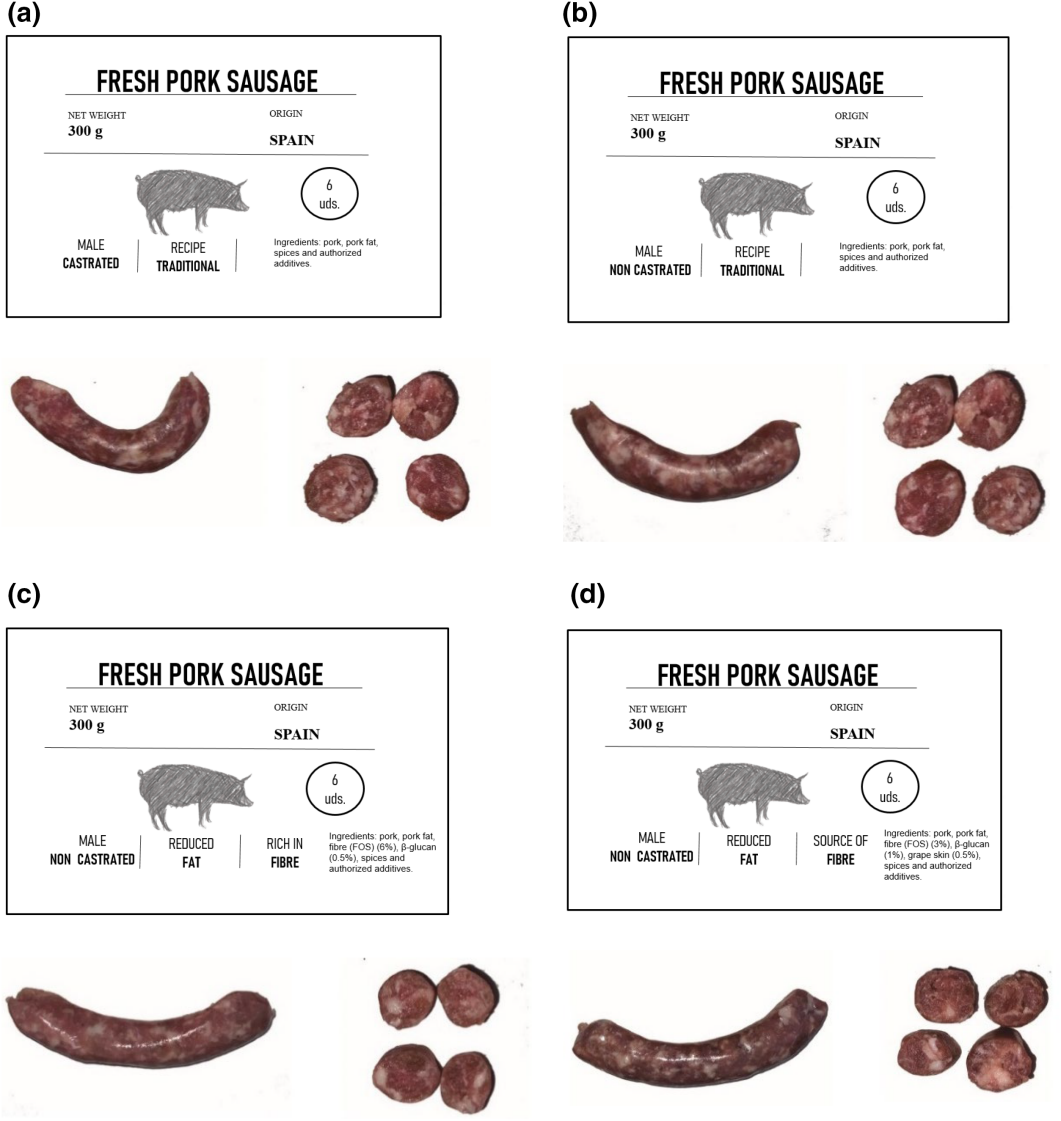
Image of the labeling of the different sausages. (a) “Castrated” sausage made from castrated pork, with 30% pork back fat, and 70% lean ham, made with a traditional recipe. (b) “Non‐castrated” sausage made from entire pork, with 30% pork back fat and 70% lean ham. (c) “Non‐castrated F6%” sausages made from non‐castrated pork, with 20% pork back fat, 6% (w/w) inulin, and 0.5% (w/w) β‐glucan. (d) “Non‐castrated F3%” sausages made from non‐castrated pork, with 20% pork back fat, 3% (w/w) inulin, 1% (w/w) β‐glucan, and 0.5% (w/w) grape skin.

The CATA test is an effective method used in other food products that could include subjective terms such as quality and price (Los et al., 2021). Consumers were asked to complete a “check all that apply” (CATA) questionnaire from the images provided (Figure 1). The terms in the questionnaire were generated from previous studies (Table 2). Sixteen terms related to sensory characteristics of fresh sausage appearance, hedonic terms and concepts on animal welfare, health and price of the products were used, eight of which corresponded to sensory characteristics and eight to concepts or hedonic terms (Table 2). Consumers had to check all the terms of the list they considered appropriate to describe each product. The order of presentation of the terms in the CATA questionnaire was randomized by groups of attributes in each sample to avoid order bias (Peñaranda et al., 2020).

**TABLE 2:** Characteristics of the survey participants.

Variables	Total (*n* = 131)	%
Gender		
Female	68	51.9
Male	63	48.1
Age group (years)		
18–30	41	31.3
31–45	24	18.3
46–60	47	35.9
More than 60	19	14.5
Level of education		
Primary	9	6.9
Secondary	42	32.1
Higher/university	80	61.1
Employment		
Inactive	36	26.5
Active	85	64.9
Retired	10	7.6
Income (per month)		
Less than the mean (<2000 €)	39	29.8
As the mean (2000–2500 €)	49	37.3
More than the mean (> 2500 €)	43	32.8
Area of residence		
Urban	116	88.5
Rural	15	11.5
Previous knowledge about castration		
Yes	39	29.8
No	92	70.2

Figure 1 shows the images used on the CATA questionnaire of all the evaluated products with their corresponding labels. Thus, labels included some fixed information, i.e., the net weight of the product (300 g), the origin (Spain), and the units in the package (6 units), as well as some variable information, i.e., the type of pig from which it was elaborated (non‐castrated or castrated), the fat content (no claim, i.e., “traditional” or “reduced”), and the fiber content (no claim, “source of fibre” or “rich in fibre”). Additionally, the recipe was included: original, rich in fiber (more than 6 g/100 g) or with a high content of fiber (more than 3 g/100 g), and the ingredients that made up each sausage. The fat and fiber claims were added following the EU Regulation (2006).

The first image (Figure 1a) corresponded to a sausage made from castrated pork, with 30% pork back fat and 70% lean ham, and with a traditional recipe. The second image (Figure 1b) was a sausage with the same formulation as in Figure 1a, but made from entire pig meat (non‐castrated male pig meat). The remaining two formulations were composed using 20% pork back fat. For Non‐castrated F6% (Figure 1c): 6% (w/w) inulin, 0.5% (w/w) β‐glucan, and for Non‐castrated F3% (Figure 1d): 3% (w/w) inulin, 1% (w/w) β‐glucan, and 0.5% (w/w) grape skin (Egea et al., 2020).

To improve the quality of the data and detect bots at the end of the survey, an inconsistent question was incorporated to check for attention. In addition, time data were collected to complete the survey with an average response time of 8 min and 05 s and consistency checks were performed on the responses to each question in the questionnaire.

### 2.3 Statistical analysis

For the statistical analysis and processing of the consumer survey data, the statistical software package SPSS 24 (SPSS, Chicago, IL, USA) was used. The frequency of “yes” or “no” answers was determined for each question. A chi‐square test was used to compare demographic parameters and the consumer habits questions.

A Shapiro–Wilk test was carried out to study the normality of the distribution of the beliefs section (scale from 1 to 9). As the samples did not have a normal distribution, a non‐parametric Kruskal–Wallis test was performed considering the sex, age, level of education, employment, income, awareness of animal welfare, and the “yes‐no” categories from section 1 (“Bad experience”, “Read information”, “pork product perception”, “Trimming fat,” and “fibre consumer”) as fixed sources of variation.

For the analysis of the CATA test, as mentioned in Peñaranda et al. (2020), the frequency of mention of each attribute was determined through the sum of the number of consumers who used that attribute to characterize each of the four sausage samples. Cochran's Q test was carried out to identify significant differences among samples for each of the attributes included in the CATA questions. To analyze data from the CATA questionnaire, in accordance with the chi‐square distance and the frequency tables, a correspondence analysis was used, and the bidimensional maps representing samples and descriptors were obtained. For that purpose, a data matrix composed of 4 rows (products) and 16 columns for the attributes was used. These data were generated for each consumer (131), indicating “0” when the attribute was not selected and “1” when it was chosen. Then, the correspondence analysis was used to analyze the associations between categorical variables in a data table. The variables were represented as points in a multidimensional space, with the distances between the points indicating the strength of the associations with the different samples studied. Only attributes with frequency above 20% were considered (Los et al., 2021). All these statistical analyses were performed using the XLSTAT 2017 package (Addinsoft, Paris, France).

## 3 RESULTS AND DISCUSSION

### 3.1 Consumers’ characteristics

Table 2 shows the characteristics of the participants. The surveyed population was composed of 48.1% women and 51.9% men, whose ages ranged between 18 and 30 years (31.3%), between 30 and 45 years (18.5%), between 46 and 60 years (35.9%), and over 60 years (14.5%), with primary education (6.9%), secondary education (32.1%), and higher/university education levels (63.6%). Younger consumers were overrepresented and older consumers were underrepresented, and this could have an effect on the results obtained, so it needs to be considered.

In relation to employment, 26.5% of the participants were inactive, 64.9% were active, and 7.6% were retired. A total of 27.5% of them had an income below the average (<€2000/month), 35.9% had an income equal to the average (between €2000 and 2500/month), and 35.2% had an income above average (>€2500/month) with respect to income values in Spain. With respect to the area where they live, 88.5% lived in an urban area and 11.5% in a rural one. The mean response time was 8 min and 05 s.

### 3.2 Consumers’ habits

Table 3 shows the results of the habits and experience questions. The percentage of people who had had an unpleasant experience when consuming pork or meat products in the last six months was 8.4%. It is possible that this bad experience was related to the presence of androstenone or skatole, as in Spain, most of the pigs produced are non‐castrated (Aluwé et al., 2020), and some consumers could be sensitive to androstenone or skatole. Androstenone is a male sex pheromone with an odor similar to that of urine or sweat and is perceived by approximately 40%–50% of consumers, while skatole is a metabolite of tryptophan associated with a fecal odor or naphthalene and is perceived by 99% of consumers (Font‐i‐Furnols, 2012). Aluwé et al. (2020) in a study from 17 countries found that around 30% of the respondents indicated to have experienced a bad smell or taste when consuming pork. A higher percentage of bad experience was expected, although it is possible that these lower values could be related to people sensitive to androstenone who stopped eating pork to avoid these bad experiences, although it is necessary to take into account that androstenone sensible consumers sometimes reduce the consumption of this product. There was a significant effect according to the age range (*p* = .010) and income (*p* = .042). In terms of age range, previous studies have shown that consumers aged 46–60 years are the most likely peope to have experienced unpleasant odors when consuming meat (Garrido et al., 2023). In terms of income range, it can be observed that those who had declared to have a medium and/or below‐average income level had a bad experience when consuming meat. Normally, people with a higher‐than‐average income level are more likely to buy ready‐to‐eat meat dishes (Caputo et al., 2018) with some kind of processing, which may have influenced the presence of boar taint. In general, the perception of boar taint varies between different pork products, as the processing of meat products decreases the risk of boar taint, due to the use of cooking, fermentation, smoking, and the use of spices (Peñaranda et al., 2017; Škrlep et al., 2020).

**TABLE 3:** Habits and experience questions: absolute frequency (and %) of each answer and significance of the demographic parameters.

Habits versus demographics			*p*‐value (Chi‐square test)						
Question	Yes	No	Gender	Age	Area of residence	Level of education	Employment	Income	Castration knowledge
Have you had any unpleasant experiences consuming pork or meat products in the last six months?	11 (8.4)	120 (91.6)	.693	.010	.797	.539	.423	.042	.490
Do you usually read the information on the food labels?	65 (49.6)	66 (50.4)	.257	.791	.161	.905	.133	.365	.219
Is your perception of pork products positive?	114 (87.7)	16 (12.2)	.141	.189	.480	.270	.335	.133	.125
Do you trim fat from meat products or meat before eating?	93 (71)	38 (29)	.186	.435	.319	.444	.665	.067	.810
Do you normally eat products rich in fibre?	90 (68.7)	41 (31.3)	.698	.036	.857	.263	.264	.884	.115

The result for the question “Do you usually read the information on the food labels?” was fairly balanced, with a total of 65 (49.6%) people answering “yes” and 66 (50.4%) “no.” In a study carried out by Rimal (2005), 80% of those surveyed considered that it was very important that meat labels contain information on nutrition, ingredients, health properties, and the production process of meat products. However, according to the Food Safety and Quality Congress (AECOC) study (2019), the information on the labels was very important for 56% of the buyers. In the case where 9 out of 10 people read the labels, the following four aspects were the most important: the expiration date, the type of animal from which the meat came from, the price, and the presence or absence of quality seals. All the demographic parameters studied were not significant.

For the question “Is your perception of pork products positive”, a total of 114 people (87.7%) had a positive perception of pork and its derivatives, and 16 people (12.3%) perceived it as bad, without a significant effect (*p* > .05) of the demographic parameters studied or previous knowledge on castration. It is important to point out that for the present study all people who did not eat meat were excluded from the sample. This favorable result was therefore to be expected, as pork is traditionally consumed in Spain, with a processed pork consumption of 11.3 kg per capita in 2021 (MAPA, 2021).

With respect to the question “Do you trim fat from meat products or meat before eating?”, the result showed that 93 people (71%) answered that they removed the fat before eating meat, while 38 people (29%) did not usually do so. This may be because consumers tend to perceive fat as not beneficial to their health or because they do not like its taste. A previous study carried out in Spain by Kallas et al. (2013) reported that 41% of consumers declared eating pork with fat. Thus, there has been an increase in the number of consumers who remove fat before eating in the last 10 years. Moreover, there is an effect of age in this answer, with younger individuals being the most likely to declare fat trimming. This is perhaps related to the increase in health concerns (Plasek et al., 2020). In addition, this trend can also be observed in the fact that a total of 90 people (69%) declared normally consuming products rich in fiber, whereas 41 people (31%) did not. With these results, it is possible to observe that the percentage of people who consumed foods rich in fiber was high, so it is expected that products rich in fiber will be perceived as someting positive. Plasek et al. (2020) indicate that: “Per the United States Food & Drug Administration (FDA), Healthy foods are defined as those that are “low in fat, low in saturated fat, contain at least 10% of the daily value for vitamins A, C, calcium, iron, protein fibre” and are limited in the amount of sodium and cholesterol (USFDA).” Positive effects on health have been associated to meat products such as frankfurters as a source of dietary fiber, because dietary fiber is associated with the good functioning of the intestine (Polizer Rocha et al., 2018). As Vidal et al. (2020) remarked, the health awareness is important for predicting a variety of attitudes and behaviors on meat products.

### 3.3 Consumer beliefs toward animal welfare and pig castration

The total population surveyed agreed with an average of 7.5, with the statement “Animal welfare worries me” (Table 4). This is a fairly high number, which indicates that, as previously highlighted, animal welfare has become a priority for modern consumers. According to the AECOC (2019) study, in recent years, there has been an increase in concern and awareness in today's society: “Consumers experience a duality of feelings regarding meat products, and this is reflected in their consumption habits”. In fact, the meat paradox is defined as the fact that consumers like animals but, at the same time, they eat animals (Herzog, 2010). Moreover, consumers seem to experience a feeling of guilt toward mistreated animals under the social pressure that eating meat is bad for health and the environment (AECOC, 2019). Even though we found an agreement with the statement “Animal welfare worries me,” the level of agreement was significantly (*p* < .05) higher in consumers who declared to read label information and to trim fat (Table 4). This information could be interesting, due to the fact that although the participant read the exposed half part of the labels, if they are aware about animals, it is possible that some statement about animal welfare or animal castration could influence them in their purchase intention. For that, it is important that the information used should be written in a simple and direct language, as remarked by Nogueira et al. (2023).

**TABLE 4:** Means of the agreement regarding statements by all the consumers and those classified according to their answers to habits and experience questions.

Statements	Total mean	*SEM*	1 Unpleasant experience		2 Read information		3 Pork product perception		4 Trimming fat		5 Fiber consumer	
			Yes	No	Yes	No	Yes	No	Yes	No	Yes	No
Animal concern												
Animal welfare worries me	7.5	0.141	7	7.6	7.9^a^	7.2^b^	7.5	7.5	7.8^a^	7^b^	7.6	7.4
I know that sometimes male pigs intended for consumption are castrated without anaesthesia at birth	3.0	0.242	4.5	2.9	2.9	3	2.9	3.7	3	3.2	2.8	3.2
I think castration of pigs without anaesthesia should not be performed	7.6	0.231	7.6	7.6	7.8	7.4	7.7	7.3	8	6.7	7.6	7.7
The castration of pigs is justified because it improves the smell and flavour of the meat	4.2	0.272	5.6	4.1	4.5	3.4	4.3	3.5	4	4.9	4.1	4.6
I believe that meat from castrated pigs should be labelled as such	7.4	0.206	7.4	7.4	8^a^	6.8^b^	7.2	8.3	7.6	6.9	7.3	7.6
Fat perception												
A high fat content in a meat product is negative for me	7.1	0.189	6.7	7.1	7	7.2	7	7.5	7.6^a^	5.7^b^	7.1	7.1

*Note*: Different superscripts (a; b) indicate significant differences (*p* < .05) between consumers' classification within habit or experience after applying the Krustal–Wallis test for independent samples. Scores from 1: ‘not at all agree’ to 9: ‘completely agree’.

Table 5 shows the effect of the demographic parameters studied and the previous knowledge about castration on the statements. Gender, age, employment, income, area of residence, and previous knowledge about castration did not show any significant effect (*p* > .05) on the statement “animal welfare worries me” (Table 5). In disagreement with our results, previous works have shown that women are more concerned about animal welfare than men (García‐Gudiño et al., 2021). Differences could be related to the inclusion criteria, as there are also more vegan women than men (Randler et al., 2021). This study also did not find differences between age groups, although it indicated that a few previous works showed that the importance of animal welfare decreased with age. People with a primary education agreed more with the statement “I am concerned about welfare” than people with a secondary education (Table 5).

**TABLE 5:** Beliefs of consumers by demographic characteristics and previous castration knowledge related to animal welfare issues and practices and fat content perception.

Attitude (*N* = 131)	Gender		Age				Education			Employment			Incomes			Area of residence		Castration knowledge	
	Female	Male	18–30	31–45	46–60	>60	P	S	U	I	A	R	Low	Mean	High	Rural	Urban	Yes	No
Animal welfare issues and practices																			
Animal welfare worries me	7.5	7.5	7.4	7.3	7.8	7.5	8.4^a^	7.2^b^	7.6^ab^	7.4	7.6	7.2	7.6	7.5	7.5	7.6	7.5	7.4	7.6
I know that sometimes male pigs intended for consumption are castrated without anaesthesia at birth	3.0	3.0	2.9	2.3	3.4	2.9	4.4	2.6	3	3.2	3.0	2.1	2.7	3.5	2.6	3.0	3.0	2.9	3.2
I think castration of pigs without anaesthesia should not be performed	7.2	8.0	8.0	7.2	7.7	7.6	8.0	7.7	7.6	8.0	7.5	7.6	7.4	7.5	7.9	7.5	7.7	7.5	7.7
The castration of pigs is justified because it improves the smell and flavour of the meat	4.6	3.9	3.3	4.0	5.0	4.4	6.9^a^	4.3^b^	3.9^b^	3.2^a^	2.1^b^	3.0^a^	3.6	4.1	4.8	6.1^a^	4.0^b^	4.9	4.0
I believe that meat from castrated pigs should be labelled as such	7.7	7.2	7.2	7.3	7.6	7.5	7.8	7.7	7.2	7.3	7.4	7.8	7.2	7.2	7.9	7.7	7.3	7.4	7.4
Fat content perception																			
A high fat content in a meat product is negative for me	7.2	7.0	6.9	7.0	7.4	7.0	7.8	7.1	7.1	7.3	7.1	6.9	7.2	7.2	6.8	6.5	7.2	7.3	7.0

*Note*: Items in the same row and within classification category with different superscripts are significantly different (*p* < .05) after the application of a Krustal–Wallis test for independent samples; P: Primary, S: Secondary, U: Higher/university; l, High; I: Inactive, A: Active, R: Retired. Bachelor's degree, Low: <2000, Mean: 2000–2500, High: >2500. Scores from 1: ‘not at all agree’ to 9: ‘completely agree’.

An average of 3 (=disagree) points were obtained for the statement “I know that sometimes male pigs intended for consumption are castrated without anaesthesia at birth.” This indicates that, as Cabana (2010) explained more than 10 years ago, most consumers did not have sufficient knowledge about production practices when making a purchase. However, this is not a unique situation in Spain, as this has also been observed in other countries. In fact, a study with 4031 consumers from Belgium, France, Germany, and the Netherlands showed that on average, 48.5% of them had never heard about physical castration and 53.7% of the consumers had never heard about boar taint (Vanhonacker & Verbeke, 2011). However, although this practice has been reduced in most EU countries, there is no evolution in the education of the population in this direction. In the present work, no information was previously given to consumers, as in the previous study by Tomasevic et al. (2020), in which the authors indicated: “this is a limitation of the study since it can affect the answer of the consumers, but on the other side, it reflects the beliefs and attitudes in the real situation, without added information.”

The statement “I think castration of pigs without anaesthesia should not be performed” obtained a score of 7.4 on average. No effect of habits or demographic characteristics on the scores of this statement was observed (Tables 4 and 5). Similar results were found by Kallas et al. (2013) in France, the United Kingdom, and Germany. Consumers from Bosnia and Herzegovina, Bulgaria, Croatia, North Macedonia, Moldova, Serbia, and Ukraine agreed with the statement “Castration causes pain to the animals” (Tomasevic et al., 2020). Castration is a painful and stressful procedure (Marchant‐Forde et al., 2009) and it is recognized as a considerable animal welfare problem. Other studies that did not include the “anaesthesia” term found different results. Font‐i‐Furnols and Guerrero (2022) observed no differences between clusters for the statement “Castration causes pain to the animals” and “Castration is necessary,” with all the scores close to the intermediate level of the agreement scale toward agreement, indicating that the participants did not have a clear opinion about this statement, or that the question was not seen either as something intrinsically good or as something intrinsically bad. Similar results were obtained for these statements by consumers from Eastern European countries, such as the Czech Republic, Poland, Slovakia, and Slovenia (Tomasevic et al., 2020). Thus, there is no consensus on this point, although, in general, both the application of anaesthesia or analgesia and the application of immunocastration were generally rated better than castration with no pain relief (Aluwé et al., 2020).

The statements “The castration of pigs is justified because it improves the smell and flavour of the meat” and “I believe that meat from castrated pigs should be labelled as such” received average scores of 4.2 and 7.4, respectively. In Cabana (2010), the answers to these questions obtained average scores of 5.82 and 6.14, respectively (in a 9‐point scale), and Kallas et al. (2013) obtained average scores of 5.6 and 8.2 (in a 9‐point scale), respectively. The same questions were asked after the sensory experience and with information on castration, and in this case, only the rating on the labeling of castrated pork was somewhat lower (7.6) (Kallas et al., 2013). An evolution of consumer opinion on these issues over the last 12 years could be observed, because it seems that there might be an increase in the importance of the respondents who think they should know the treatment given to the pigs during their production. In relation to further castrating due to improved sensory quality, the consumer opinions were very different; they may believe that it is too painful for the animals if it is not done properly, and this is important, since castration (without anesthesia) is still a common practice in many countries (Aluwé et al., 2020). In this regard, the research by Kallas et al. (2012) presents results on the relative importance of castration in animal welfare in six European countries. Although the results differed from country to country, the relationship between “no castration” and animal welfare received the lowest scores (in comparison to other questions concerning castration).

Although the consumers had general knowledge about the item “I know that sometimes male pigs intended for consumption are castrated without anaesthesia at birth,” it must be pointed out that in García‐Gudiño et al.'s (2021) work, consumers neither agreed nor disagreed (average score of 3.0) regarding the use of castration. Other studies in Europe observed that even though the surgical castration of piglets was criticized because of animal welfare issues, a low importance was found on castration in consumers’ purchasing intention or worries (García‐Gudiño et al., 2021; Kallas et al., 2013; Tomasevic et al., 2020). Even though an effect of demographic characteristics was not found on this statement in the present study. The item “The castration of pigs is justified because it improves the smell and flavour of the meat” received higher agreement scores from people with a lower level of education, as compared to those with a higher level of education (*p* < .05). Individuals who lived in rural areas also scored this statement highly (*p* < .05), perhaps because they are more aware of production practices and their reason for giving this score is due to their proximity to the production areas. So, if the market would add a label to remark that animals are not castrated, it is possible that the consumer does not appreciate this information, due to the lack of knowledge about this topic. Nogueira et al. (2023) concluded in their study that technical terminology does not have a positive effect on the participants. So, perhaps, terms such as “animal welfare” should be better options, since there is a more consistent response among participants regarding this term.

### 3.4 Consumer beliefs toward fat content and correlation between statements

With a global average score of 7.1 ± 2.17 (data not included in Table 5), the total population surveyed considered that a product with high content of fat was negative for them. This negative perception can be observed in the increase in the consumption of fat‐free products, as demonstrated in the study by Cabana (2010), where out of 94% of the people who ate meat, 59.6% claimed to consume fat‐free pork, while 40.4% preferred pork with fat. Thus, there is an increasing interest in developing meat products with healthier attributes (Polizer Rocha et al., 2018). Gender, age range, level of education, income level, employment, area of residence, and previous knowledge about castration showed no significant (*p* > .05) effects on fat perception.

Table 6 shows the Pearson correlation coefficients between the different statements studied. A correlation was found between “Animal welfare worries me” and “I think castration of pigs without anaesthesia should not be performed,” “I believe that meat from castrated pigs should be labelled as such,” and the negative perception of fat. However, even though it was significant, it was also very low, and thus not relevant. The strongest correlation (*r* = .44) was found between the previous knowledge of the castration of pigs and the fact that it could be justified by its favorable effect on odor and flavor. Other studies remarked that the perception of animal welfare may be influenced by the level of knowledge (Pejman et al., 2019). A lack of knowledge about a management practice can produce a more negative reaction from consumers toward this practice. Thus, non‐connoisseurs of practices, such as castration, may view them negatively, because they do not know either that these practices are routinely performed or the reason behind them (García‐Gudiño et al., 2021).

**TABLE 6:** Pearson's correlation coefficients between the beliefs and statements of Spanish consumers about castration practice, welfare, and fat content.

	1	2	3	4	5	6
1. Animal welfare worries me	1					
2. I know that sometimes male pigs intended for consumption are castrated without anaesthesia at birth	0.150	1				
3. I think castration of pigs without anaesthesia should not be performed	0.191*	0.005	1			
4. The castration of pigs is justified because it improves the smell and flavour of the meat	−0.015	0.435**	−0.030	1		
5. I believe that meat from castrated pigs should be labelled as such	0.328**	−0.083	0.344**	−0.080	1	
6. A high fat content in a meat product is negative for me	0.226**	−0.092	0.346**	−0.382**	0.262**	1

** *p* ≤ .01;

* *p* ≤ .05.

Finally, people who stated that the fat content was negative also agreed with the fact that castration without anesthesia should not be performed, and that the labeling of meat from castrated pigs should be required, but did not agree with the justification of castration to improve odor and flavor. It has been observed that consumer behavior is multidisciplinary, in which many factors are involved, not only the intrinsic characteristics of the product, but also external factors such as psychosocial, ethical, animal welfare, environmental impact, and sustainability (De Araújo et al., 2022; Font‐i‐Furnols & Guerrero, 2014). Consumers increasingly demand healthier, safer, and environmentally friendly products with specific rules that meet their needs (Font‐i‐Furnols & Guerrero, 2014; Ruiz‐Capillas et al., 2021). Furthermore, they perceive animal welfare as a positive factor when purchasing a meat product (Tomasevic et al., 2022). However, if this is detrimental to the intrinsic characteristics of the product, such as the presence of boar taint, they grant priority to the sensory quality of the product, as they do not want to see its sensory qualities diminished (Ruiz‐Capillas et al., 2021).

### 3.5 Check‐all‐that‐apply

Table 7 shows the results obtained through the “Cochran *Q* Test” that was used to assess whether there were significant differences between the different sausages used in the survey, and the attributes that consumers chose for each one. It is possible to see that there were significant differences between types of sausages for all attributes and terms evaluated (*p* < .05), except for the attribute “Bright.” However, the “bright” attribute was chosen more than 30 times for three out of four sausages, and 24 for the remaining one. This might indicate the importance of this characteristic. In the physical study of the same samples, Egea et al. (2020) observed that there was a reduction in lightness in both the products with fat reduction and enriched with fiber (L*, control 54.3 vs. non‐castrated F6% 46.6 and non‐castrated F3% 41.5). Color is a very interesting parameter for cooked meat products, as consumers associate this type of meat product with a bright and characteristic pink color (product‐specific) (Šojić et al., 2011). Morin et al. (2002) found that consumers preferred low brightness in meat and meat products. This was apparently due to consumers associating lighter colors with higher fat content, or it may simply be that they did not like lighter sausages as much.

**TABLE 7:** Frequency of mentioned attributes for the four different sausage samples.

Attributes	*p*‐value	Castrated	Non‐castrated	Non‐castrated F6%	Non‐castrated F3%
Dark	.009	20^b^	12^ab^	6^a^	8^a^
Bright	.274	36	33	35	24
Animal cruelty	.001	40^b^	17^a^	13^a^	32^b^
Dry	.001	17^b^	34^c^	10^ab^	5^a^
Fatty	.001	84^d^	45^c^	5^a^	24^b^
Light	.001	11^a^	9^a^	29^b^	26^b^
Matte	.001	8^a^	43^b^	5^a^	41^b^
Healthy	.001	12^a^	62^b^	13^a^	12^a^
Juiciness	.001	52^c^	8^a^	35^b^	30^b^
Appetizing	.001	59^c^	15^a^	32^b^	16^a^
Little fat	.003	11^a^	33^b^	22^ab^	29^b^
Unhealthy	.001	50^b^	8^a^	13^a^	17^a^
Expensive	.001	4^a^	19^b^	48^c^	33^bc^
Animal friendly	.001	4^a^	11^ab^	23^bc^	25^c^
Unpleasant	.001	15^a^	8^a^	35^b^	41^b^
Cheap	.001	27^b^	12^a^	34^b^	7^a^

*Note*: Castrated: sausage made from castrated pork, with 30% pork back fat, and 70% lean ham, made with a traditional recipe. Non‐castrated: sausage made from entire, non‐castrated pork, with 30% pork back fat and 70% lean ham. Non‐castrated F6%: sausages made from non‐castrated pork, with 20% pork back fat, 6% (w/w) inulin, and 0.5% (w/w) β‐glucan. Non‐castrated F3%: sausages made from non‐castrated pork, with 20% pork back fat, 3% (w/w) inulin, 1% (w/w) β‐glucan, and 0.5% (w/w) grape skin. Different superscripts (a; b; c) between products indicate significant (*p* ≤ .05) differences according to Cochran's *Q* test.

**FIGURE 2:**
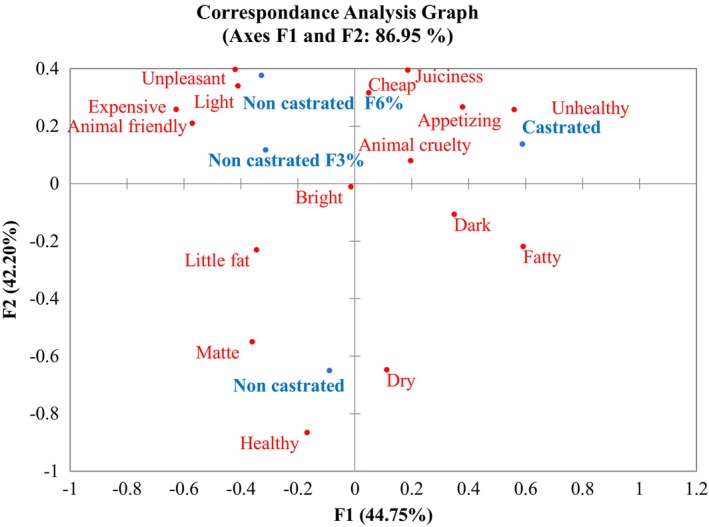
Representation of the average of sausage samples and the correlation of the attributes selected by 131 consumers in the first and second dimensions. Castrated: sausage made from castrated pork, with 30% pork back fat, and 70% lean ham, made with a traditional recipe. Non‐castrated: sausage made from entire, non‐castrated pork, with 30% pork back fat and 70% lean ham. Non‐castrated F6%: sausages made from non‐castrated pork, with 20% pork back fat, 6% (w/w) inulin, and 0.5% (w/w) β‐glucan. Non‐castrated F3%: sausages made from non‐castrated pork, with 20% pork back fat, 3% (w/w) inulin, 1% (w/w) β‐glucan, and 0.5% (w/w) grape skin.

To visualize the relationship between all the attributes and the different types of sausages, a correspondence analysis was performed in accordance with the CATA contingency table (Table 7) that produced a two‐dimensional (2D) map (Figure 2). The first and second dimensions explained 86.95% of the variance of the results (44.75% and 42.20%, respectively) allowing us to differentiate three groups. The first group consisted of the Castrated sample, a sausage elaborated with the traditional recipe, which was mainly related to the attributes and terms “fatty,” “juiciness,” “animal cruelty,” “appetizing,” “unhealthy,” “cheap,” and “dark.”

The term “fatty” was first associated with the castrated group, followed by the non‐castrated, and logically, it was negatively associated with the fat‐reduced products. One of the advantages of castration is that animals tend to have a higher fat content, and therefore more juicy meat (Škrlep et al., 2020). The association with “fatty” could be related to the “appetizing” term that was also importantly associated with sausages from castrated pigs. Meier‐Dinkel et al. (2016) carried out a study of consumers’ perception and acceptance of boiled and fermented sausage, in which fat from an entire male pig was used in different percentages (0%, 50%, and 100%), and also found that the 100% group was also associated to the term “fatty.” Also, “unhealthy” was associated with the castrated group, probably due to the “fatty” association with this product. These results are similar to those found by Polizer Rocha et al. (2018), who remarked that there was a negative association between traditional frankfurters being unhealthy, fat/calories, and high blood pressure, and also, that the category “light/low calorie” was the second‐most mentioned for the stimulus “frankfurter with fat reduction.” Therefore, in that study, it was possible to consider that the category “healthiness” was relevant for the consumer. The results obtained by Dean et al. (2012), after an evaluation of the influence and perception of consumers regarding nutritional claims, suggested the hypothesis that when people are motivated by these claims, they are susceptible to being influenced. Moreover, they tend to prefer claims that describe a reduction in the risk of common diseases related to lifestyle, rather than claims that promote benefits to their health. The present results also showed that “animal cruelty” and “cheap” were associated with castrated animals. Similar results were found by Aluwé et al. (2020) in an exploratory survey on European consumer and stakeholder attitudes toward alternatives to surgical castration of piglets, because castrating pigs was defined by consumers as a cruel practice to animals, stressful, and which produced cheap (low quality) meat.

The second group consisted of the sausages from non‐castrated male pig meat, elaborated with a traditional recipe associated to the attributes “healthy,” “matte,” “dry,” and “little fat.” In the work by Aluwé et al. (2020), the entire male meat was also associated with natural (less greasy and healthier), cheap, and bad taste. In previous studies where Frankfurt sausages were analyzed by consumers, the category “healthiness” was the most frequently mentioned for the stimuli (kinds of frankfurter) with healthier attributes (Polizer Rocha et al., 2018). In the present study, sausages had no health claims.

The third group was comprised by the reduced samples (non‐castrated F6% and F3%), and both sausages showed a similar behavior, characterized by the following terms: “expensive,” “light,” “little fat,” “animal friendly,” and “unpleasant.” And to a lesser extent, these samples were associated to the “juiciness” attribute. This confirms that the consumer could perceive the results found by Egea et al.'s (2020) study, where a trained panel evaluated these products, and no differences were found for this attribute between sausages with normal fat levels and the fat reduced with vegetable fibers. It is not clear if the “healthy” term was associated with human or with animal one. There was a correlation between “unhealthy” and “fatty” of 0,402, but there was no clear relation between “healthy” and “less fat.” It was expected that fat‐reduced, and high and rich in fiber descriptions would be associated to this term. Although some consumers rely on the fat and fiber content of a product for its perceived healthiness (Rizk & Treat, 2015), theclear association between fat‐reduced products with higher levels of fiber was not observed. Tobin et al. (2014) found that most of the 548 consumers of meat products confirmed that they did not consider frankfurters to be healthy products, mainly because of the high quantity of harmful chemical substances, fat, and salt. In addition, Plasek et al. (2020), in a study on the impact of extrinsic product attributes on demonstrating the healthiness of functional food products, observed that in order of importance, health claims/nutritional claims took the fifth place out of six elements, and only the nutritional claim showed a significant effect, while the tested health claim did not. It seems that consumers may be skeptical about these claims and that health claims have only a small influence on perceptions of healthiness.

An association of non‐castrated F3% with “dark” was expected, but instead, this attribute was associated with “matte.” Non‐castrated F3% contains grape fibers that could have flavonoids. These molecules are primarily involved in the color of grape pomace, and it must be considered that the color of anthocyanins could vary from red to blue depending on the pH value. In an acidic medium, anthocyanins turn red and in a basic medium, blue (Mainente et al., 2019), what could be perceived as a less bright sample.

In addition, sample F6% was associated with “cheap,” although in this study, no price was considered. In this sense, Scudino et al. (2023) observed that the attitude statements suggested the critical role of the “price” factor in consumers’ perception. In other studies, more expensive prices were associated with a frankfurter with natural antioxidants and a frankfurter with omega‐3 fatty acids (Polizer Rocha et al., 2018). The price has an impact on consumers' choices with respect to healthier products (Font‐i‐Furnols & Guerrero, 2014). Also, consumers' perceptions regarding health are crucial in determining the acceptance of reformulations toward healthier foods (Ares et al., 2008; Barreiro‐Hurlé et al., 2010). One important factor linked to consumers' judgment of the claims about healthier products is familiarity with a functional component—that is, with its health benefits (Giacalone et al., 2015; Polizer Rocha et al., 2018).

## 4 CONCLUSIONS

Animal welfare has been gaining great importance from consumers in recent years, perhaps because they care about the state of the animals and the quality of their products. Additionally, the practice of castration is important for the consumer, but there is still great ignorance about this practice by a greater part of the population, as animal welfare concerns increased when consumers were provided with additional information on this type of meat. However, there is no clear position about the fact that if castration is justified whether it would improve the smell and taste of the entire male pork, being more approved by consumers with primary education level and those who live in rural areas.

Most consumers perceive meat products as positive, although most of them have high‐fat content and they affirm that usually used to trim the fat before they consume. The evaluation of the four different products reflects that fresh sausages are associated with “animal cruelty” or “animal friendly” in relation to the castrated or not castrated label. The high content of fat is one of the factors that is related to the possible “unhealthy” term, fat reduction and fiber addition were not enough to have a “healthy association” in these kind of products, which in addition are related to an “unpleasant” term. Although reduced‐fat entire male pork sausages with vegetable fiber could be a good option for marketing, uncastrated male pork will need more than fiber claims to be associated with healthy products and the consumer should be previously informed if he could appreciate the meaning of castration labeling.

## AUTHOR CONTRIBUTIONS

**Macarena Egea:** Conceptualization (equal); data curation (equal); formal analysis (equal); investigation (equal); methodology (equal); validation (equal); visualization (equal); writing – original draft (equal). **M. Dolores Garrido:** Conceptualization (equal); formal analysis (equal); funding acquisition (equal); investigation (equal); methodology (equal); project administration (equal); resources (equal); supervision (equal); validation (equal); writing – review and editing (equal). **Maria Font‐i‐Furnols:** Conceptualization (equal); investigation (equal); methodology (equal); validation (equal); visualization (equal); writing – review and editing (equal). **Nuria Panella‐Riera:** Conceptualization (equal); investigation (equal); methodology (equal); validation (equal); visualization (equal); writing – review and editing (equal). **M. Belén Linares:** Conceptualization (equal); data curation (equal); formal analysis (equal); investigation (equal); methodology (equal); visualization (equal); writing – review and editing (equal). **Irene Peñaranda:** Conceptualization (equal); data curation (equal); formal analysis (equal); investigation (equal); methodology (equal); validation (equal); visualization (equal); writing – original draft (equal).

## FUNDING INFORMATION

Acknowledgments to the Ministry of Sciences and Innovation (MCI (Ministerio de Ciencias e innovación: RTA 2017‐00039‐02‐02)), State Investigation Agency (AEI (Agencia Estatal de Investigación)), and National Agricultural Research Institute (INIA (Instituto Nacional de Investigación Agraria)) of Spain for their financial support.

## CONFLICT OF INTEREST STATEMENT

The authors declare that they have no known competing financial interests or personal relationships that could have appeared to influence the work reported in this paper.

## ETHICS STATEMENT

Ethical approval for the involvement of human subjects in this study was granted by Murcia University Research Ethics Committee, Reference number ethics ID: 3566/2021, 13/10/2021. Participants gave informed consent via the statement “I am aware that my responses are confidential, and I agree to participate in this survey” where an affirmative reply was required to enter the survey. They were able to withdraw from the survey at any time without giving any reason. The products tested were safe for consumption.

## Data Availability

Data will be made available on request.
